# Genome-Wide Analysis of SIMILAR TO RCD ONE (SRO) Family Revealed Their Roles in Abiotic Stress in Poplar

**DOI:** 10.3390/ijms24044146

**Published:** 2023-02-19

**Authors:** Yuting Wang, Ruiqi Wang, Yue Yu, Yongmei Gu, Shuang Wang, Shixian Liao, Xiaoya Xu, Tingbo Jiang, Wenjing Yao

**Affiliations:** 1 State Key Laboratory of Tree Genetics and Breeding, Northeast Forestry University, Harbin 150040, China; 2 Co-Innovation Center for Sustainable Forestry in Southern China/Bamboo Research Institute, Nanjing Forestry University, 159 Longpan Road, Nanjing 210037, China

**Keywords:** poplar, SRO family, expression patterns, abiotic stress, RNA-Seq

## Abstract

SIMILAR TO RCD ONE (SRO) gene family is a small plant-specific gene family responsible for growth, development, and stress responses. In particular, it plays a vital role in responding to abiotic stresses such as salt, drought, and heavy metals. Poplar SROs are rarely reported to date. In this study, a total of nine SRO genes were identified from *Populus simonii × Populus nigra*, which are more similar to dicotyledon SRO members. According to phylogenetic analysis, the nine *PtSROs* can be divided into two groups, and the members in the same cluster have a similar structure. There were some cis-regulatory elements related to abiotic stress response and hormone-induced factors identified in the promoter regions of *PtSROs* members. Subcellular localization and transcriptional activation activity of *PtSRO* members revealed a consistent expression profile of the genes with similar structural profiles. In addition, both RT-qPCR and RNA-Seq results indicated that PtSRO members responded to PEG-6000, NaCl, and ABA stress in the roots and leaves of *Populus simonii × Populus nigra*. The PtSRO genes displayed different expression patterns and peaked at different time points in the two tissues, which was more significant in the leaves. Among them, *PtSRO1c* and *PtSRO2c* were more prominent in response to abiotic stress. Furthermore, protein interaction prediction showed that the nine PtSROs might interact with a broad range of transcription factors (TFs) involved in stress responses. In conclusion, the study provides a solid basis for functional analysis of the SRO gene family in abiotic stress responses in poplar.

## 1. Introduction

With continuous changes in climate environment, plants have evolved various and complex molecular mechanisms to adapt to various abiotic stresses [1]. Plant-specific genes are important for maintaining plant identity, adapting to environmental changes, and supporting plant growth [2]. To date, many plant-specific genes have been identified from different plant species, while their biological functions are still unclear. The SRO family is one of the plant-specific protein families known for playing important roles in plant growth and development and stress resistance. Particularly, the SRO family plays an important role in response to abiotic stresses such as salt and drought [3]. In addition to a poly (ADP-ribose) polymerase catalytic center (PARP, PS51059), SROs usually contain the RCD1-SRO-TAF4 structural domain (RST, PF12174) that interacts with other transcription factors (TFs) [4]. Some SROs also contain the N-terminal WWE (PS50918) structural domain, which is involved in ADP ribosylation (ADPRylation) and protein ubiquitination [5]. The SRO family is generally divided into two classes: Class I comprises WWE, PARP, and RST structural domains, while Class II comprises RARP and RST [6].

At present, our restricted insight into the SRO family mainly comes from Arabidopsis, which contains six SRO members (*AtRCD1* and *AtSRO1-AtSRO5*) [7]. AtSRO1 and AtRCD1 are homologous proteins with the same structural domain, which share the conserved domains of PARR and RST and a WWE domain at the N-terminal [8]. Different stress conditions result in partial functional redundancy of AtSRO1 and AtRCD1 [9]. AtRCD1 can interact with TFs, thereby involved in drought response mediated by the abscisic acid (ABA) signaling pathway [10]. Aside from regulating plant development, it participates in hormone signaling pathways, including ABA, ethylene (ETH), and methyl jasmonate (MeJA) [11]. There was an increase in sensitivity to salt and osmotic stresses in loss-of-function mutants of *AtRCD1* [12]. Arabidopsis double mutants showed severe defects in embryonic development and exhibited pleiotropic phenotypes with dwarf plants and short roots [13]. Intense light, salt treatment, and ozone stress can increase the expression level of *AtSRO2* and *AtSRO3*. To date, *AtSRO4* may be involved in transduction for stress, and *AtSRO5* is required for the oxidative stress response and in response to salt treatment [13,14]. There have been extensive studies of SROs in other plant species [15]. For example, SNAC1 and DST interact directly with OsSRO1c in rice to regulate stomatal closure and accumulate hydrogen peroxide in response to drought and oxidative stress [16]. OsSRO1a can negatively regulate rice resistance to bacterial blight through the jasmonic acid (JA) signaling pathway mediated by OsMYC2 [17]. *TaSRO1* promoted wheat growth and improved tolerance to multiple abiotic stresses by controlling redox homology [18]. Salt, cadmium, and oxidative stress tolerance increased in *Arabidopsis* with *ZmSRO1b* overexpression [19]. By disrupting the MYB-bHLH-WD40 (MYB-bHLHWD40) pathway, overexpression of *ZmSRO1e* in maize and Arabidopsis showed increased sensitivity to oxidative and salt stresses [19]. In apples, *MdRCD1* can positively regulate drought stress through the ABA signaling pathway [20]. A similar sequence of AtSRO5 was found in tomato, SolySRO1, and its expression was increased during salt stress [21]. *MaSROs* in bananas positively responded to biotic and abiotic stresses by forming a complex network with hormone interactions [22]. Through its PARP structural domain, MaSRO4 can regulate downstream signaling pathways that are downstream of MaNAC6 and MaMYB4 [23]. Increasing evidence suggests that SRO family members are transcriptional regulators involved in plant responses to various stresses [24]. However, there is no systematic analysis of the expression patterns of SRO family members in response to abiotic stresses in poplar.

In our research, we identified a total of nine SRO genes from poplar and determined their evolutionary relationships based on gene structure and homologous covariance analysis. We analyzed their protein sequence, chromosome distribution, and cross-species covariance. A transcriptome-based analysis of poplar leaves was conducted under salt stress, which revealed the expression pattern of the nine SRO genes. Moreover, we verified their subcellular localization and validated their transcriptional activation activity. This study provides references for the studies on gene evolution and biological functions of SRO family genes in poplar.

## 2. Results

### 2.1. Phylogenetic Analysis of SRO Family

**Figure 1:**
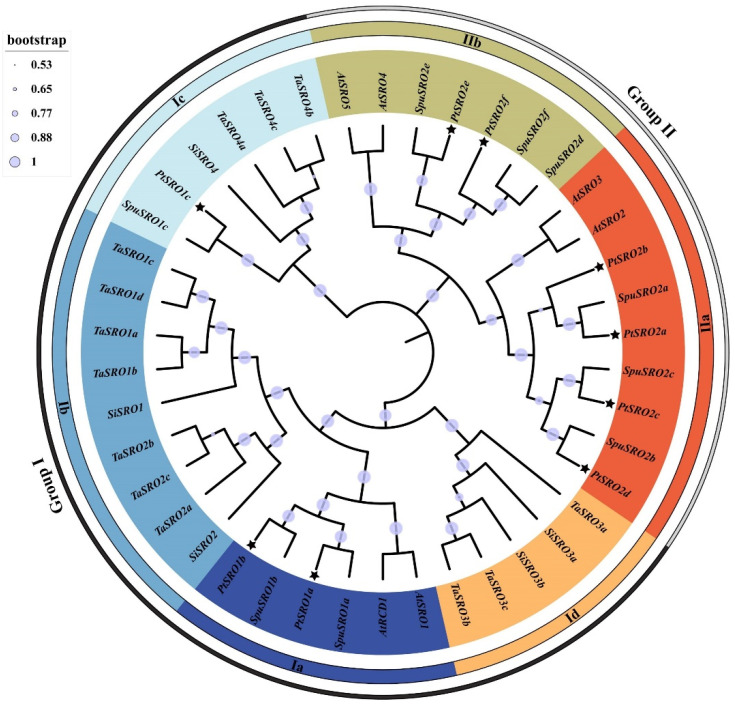
Phylogenetic analysis of the SRO proteins from poplar, Arabidopsis, Salix purpurea, Triticum aestivum, and Setaria italica. The phylogenetic tree was constructed using the neighbor-joining method in MEGA5.0 software. The evolutionary tree was divided into 6 groups; each color represents one group. Stars are marked with PtSROs.

We identified a total of nine SRO family members (E-value < 1 × 10^−5^) from poplar by HMMER, which were named PtSRO (1a–1c,2a–2f) according to their homologous gene in Arabidopsis thaliana [25]. A phylogenetic tree of SRO family genes from poplar, Arabidopsis, Salix purpurea, Setaria italica, and Triticum aestivum was constructed using MEGA5.0 [26]. As shown in Figure 1, the SRO family members from the five plant species can be divided into two groups, Group I and Group II. Group I contains four clusters, including Ia to Id, while Group II contains two clusters, including II a and II b. Group I contained SRO family members from both monocotyledons and dicotyledons, while Group II contained SROs from dicotyledons. In Group I, Ia contains family members from dicotyledons; Ib and Id contain SROs from monocotyledons, while group Ic includes SROs from both monocotyledons and dicotyledons. It indicates that the SRO gene family is more evolved in monocotyledonous plants. All the SROs from poplar and Salix purpurea were found to be in the same branch. This indicates that the two species are more closely related, and SROs diverged with the evolution of the herbaceous woody plant. In addition, we found that all genes from all species in Ia contained the full three structural domains of SRO (Table S1), suggesting that this cluster of genes may perform a wider range of biological functions than others.

We also characterized the physicochemical properties of the nine SRO proteins (Table S2). The largest amino acid sequence encoded was PtSRO1b (623), with a molecular weight of 69.23 kDa, while PtSRO2d encoded the smallest amino acid sequence (330) with a molecular weight of 36.32 kDa. Instabilities coefficients ranged from 76.09 to 86.51, while their theoretical isoelectric points (PIs) ranged from 6.43 to 9.17. The maximum hydrophilic mean values were less than 0, indicating all PtSROs were hydrophilic proteins.

**Figure 2:**
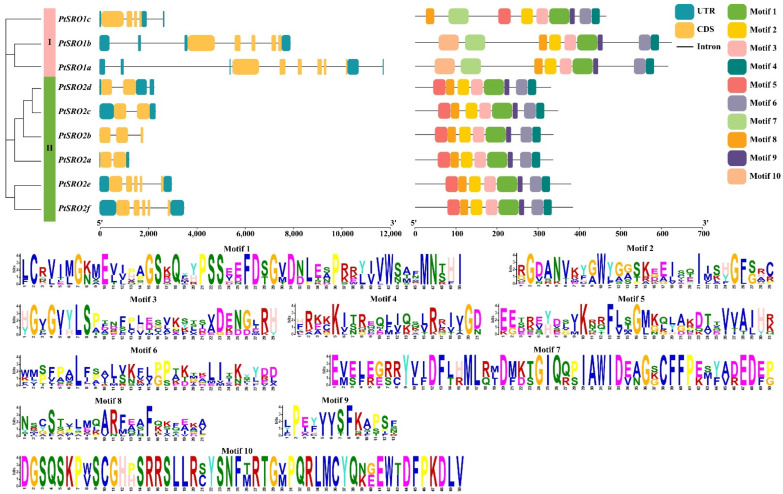
DNA structures and protein motifs of the SRO gene family in poplar. A. Gene structures. 5′ UTR and 3′ UTR are displayed using cyan bars. Black dotted lines denote introns. Colorful boxes delineate different motifs. The clustering was performed according to phylogenetic analysis. B. Visualization of multiple sequence alignment of DNA binding domains of poplar SRO members. At each position, the height of the letter piles suggests that the sequence has been conserved.

In addition, we analyzed their genetic structure using GSDS and MME online software. In general, the SRO genes that are in the same evolutionary branch have structural similarities. As shown in Figure 2, SRO2a–d belonged to Group II-a with 2–3 exons. SRO2e and SRO2f belonged to Group IIb with five exons. SRO1a and SRO1b belonged to Group Ia, with five and six exons, respectively. SRO1c belonged to Group Ic, with four exons. This indicates that the number of gene exons within each subgroup is close. Motif analysis reveals that all SROs in Group II contain the same motif and are similarly distributed. This indicates that all SROs in this group have strong similarities in gene structure. We also found that the SROs in Group I were also structurally similar. Overall, this corresponds to the results of our evolutionary tree and structural domain analysis.

### 2.2. Chromosome Distribution and Synthesis Analysis of PtSROs

**Figure 3:**
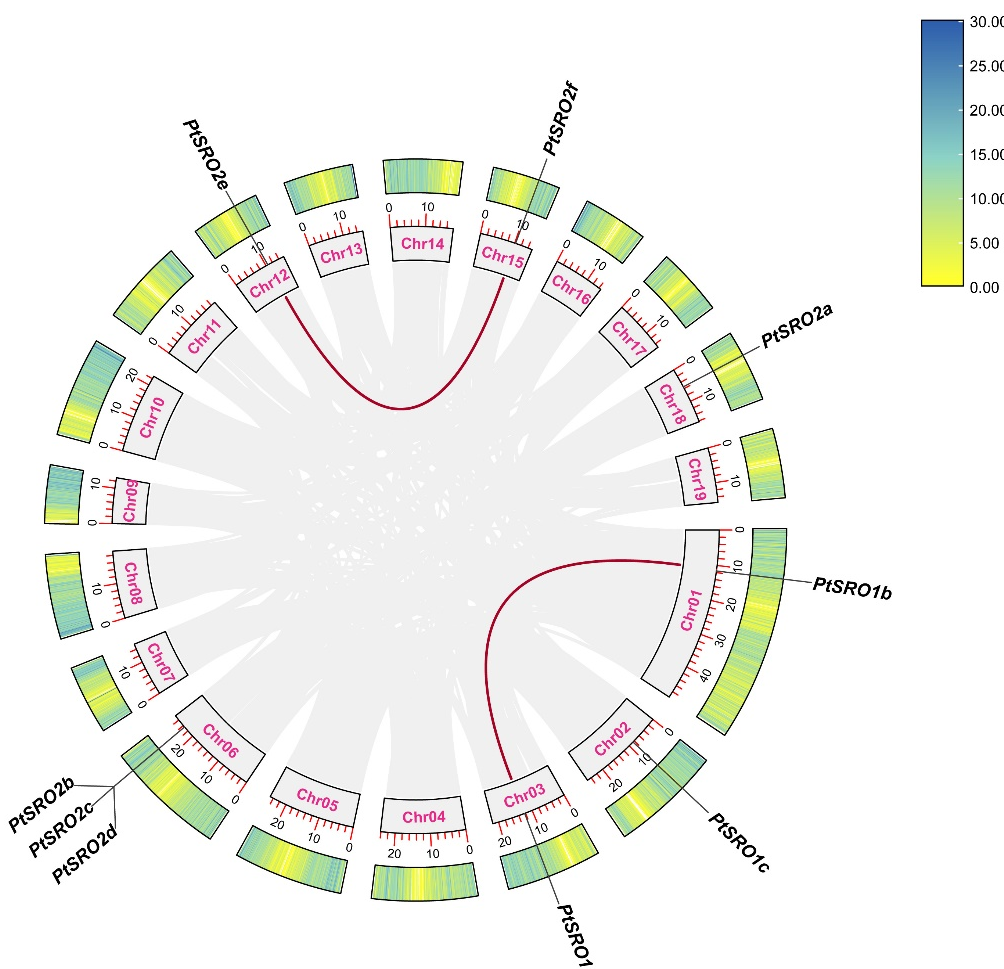
Collinearity analysis of the SRO gene family in poplar. Spectral and thermal profiles in the outer circles indicate gene density on chromosomes, with red and grey lines indicating fragment repeat gene pairs and co-linear blocks of SRO genes in the poplar genome, respectively.

We mapped SRO family genes to poplar chromosomes according to genome annotation. The nine *PtSROs* were randomly assigned to different chromosomes. PtSRO2a, PtSRO2e, PtSRO2f, PtSRO1a, PtSRO1b, and PtSRO1c were assigned to chromosomes 18, 12, 15, 3, 1, and 2, respectively. PtSRO2b, PtSRO2c, and PtSRO2d were assigned to chromosome 6. Gene duplication events were analyzed using MCScanX [27,28]. A total of two highly homologous gene pairs were obtained, both of which were tandem duplications (Figure 3). The Ka/Ks of these genes were both less than 1, indicating that strong purifying selection was experienced (Table 1). Homozygous identification of SRO1b and SRO1a was more than 80%, and SRO2e and SRO2f shared more than 90% homology, which results from the evolution of gene copy events to form a paralogous gene. 

**Figure 4:**
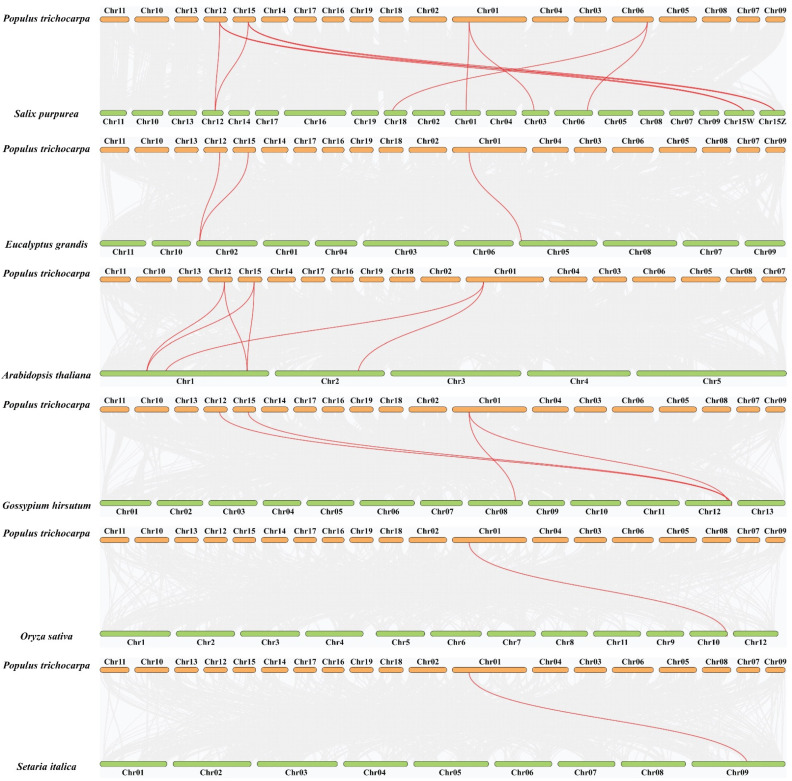
Collinearity analysis of the SRO genes from poplar and six other species. Poplar and other species share syntenic gene pairs in red and share orthologous genes in gray.

To further investigate the evolutionary relationship of PtSROs, we constructed a phylogram of SRO genes between poplar and six other plant species, including four dicots (*E. grandis*, *S. purpurea*, *G. hirsutum*, and *Arabidopsis*) and two monocots (*S. italicas* and *O. sativa*). As shown in Figure 4 and Table S3, there were 8, 3, 6, 4, 1, and 1 homologous pairs between poplar and *S. purpurea*, *E. grandis*, *Arabidopsis*, *G. hirsutum*, *O. sativa*, and *S. italicas*, respectively. Four *PtSROs* (*PtSRO1b*, *PtSRO2d*, *PtSRO2e*, *PtSRO2f*) showed a high level of collinearity with six other species. In particular, PtSRO1b had homology pairs with SRO genes from all six species.

### 2.3. Cis-Elements Analysis in the Promoter Regions of PtSROs

**Figure 5:**
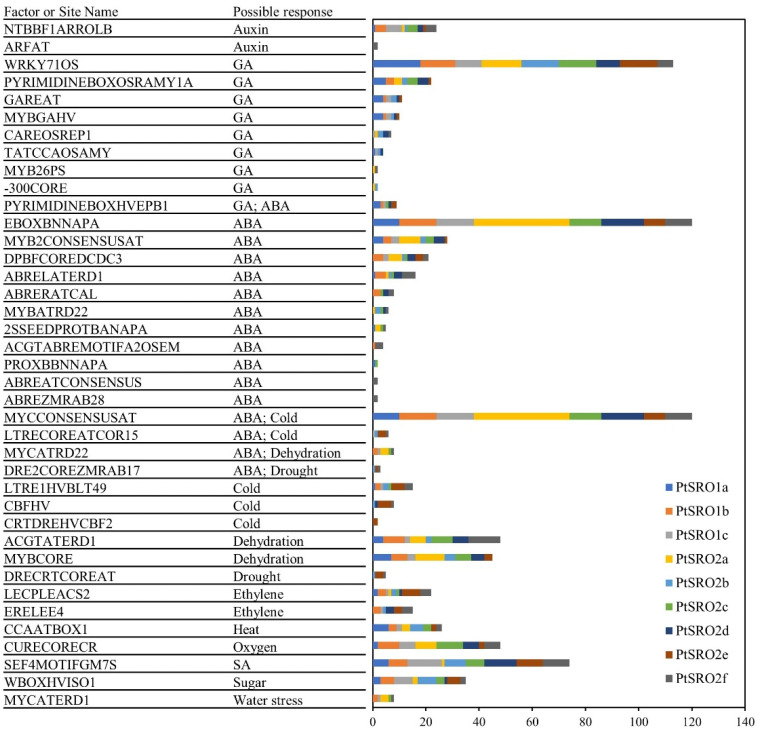
Cis-elements analysis of poplar SRO gene promoters. Different colors represent different genes.

In this study, the cis-elements in the promoters of nine PtSROs were analyzed by PLACE [29]. As shown in Figure 5 and Table S4, the elements associated with stress response covered a wide range of family members. For example, four cis-acting elements related to resistance, such as drought response, low-temperature regulation, osmotic stress, and response to dehydration, were identified in the upstream sequence of most PtSROs. There were a total of 29 phytohormone-related elements identified, including acid response elements (ABRELATERD1, ABREATCONSENSUS, ABREZMRAB28, ABRERATCAL, etc.), salicylic acid response elements SEF4MOTIFGM7S, gibberellin response element (WRKY71OS, PYRIMIDINEBOXOSRAMY1A, GAREAT, MYBGAHV, etc.), ethylene response elements LECPLEACS2 and ERELEE4, auxin response elements NTBBF1ARROLB and ARFAT. The promoters of PtSRO2b and PtSRO2e contained the least elements, with 65 and 91 elements, respectively. Each PtSROs promoter contained abundant abscisic acid (ABA) response elements; for example, PtSRO2a contained 127 ABA-related elements with maximum magnitude. Elements associated with osmosis and drought stress were also widely distributed in PtSROs promoters. All these results indicate that PtSROs participate in plant growth and development and stress responses.

### 2.4. Expression Patterns of PtSROs in Leaves and Roots under Salt Stress

**Figure 6:**
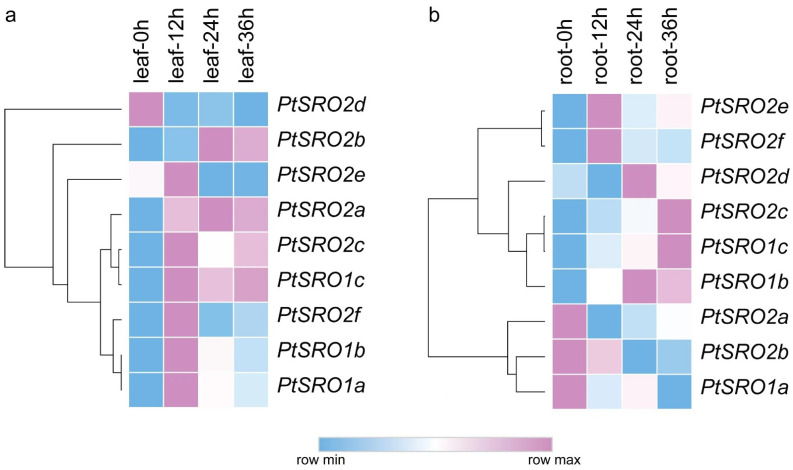
Expression pattern of poplar SRO genes. Pink represents high expression, and blue represents low expression. (**a**): Expression patterns of PtSROs in leaves at different time points under salt stress. (**b**): Expression patterns of PtSROs in roots at different time points under salt stress.

**Figure 7:**
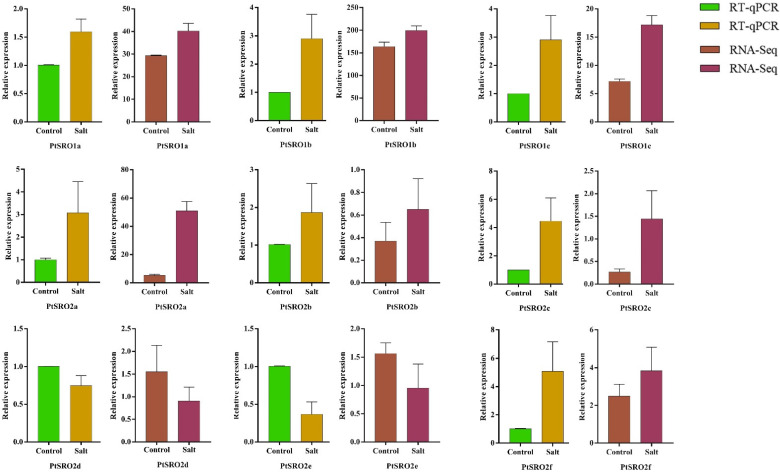
RNA-Seq and RT-qPCR were used to detect gene expression levels in leaves.

To explore the expression pattern of SROs in poplar, we profiled their mRNA abundance in leaves and roots under salt stress at different time points by RNA-Seq. As shown in Figure 6 and Supplementary Table S5, the nine genes displayed different expression patterns in the two tissues. In particular, the three PtSROs, including PtSRO1c, PtSRO2c, and PtSRO2a, were up-regulated in the leaves under salt stress, with maximum expression level at 12/24 h time points. However, the three PtSROs displayed different expression patterns in the roots. PtSRO2d was down-regulated in the leaves under salt stress, and its expression decreased with treatment. PtSRO2b was also up-regulated, and its expression reached a peak at 24 h, while the relative expression level of PtSRO1a, PtSRO1b, PtSRO2e, and PtSRO2f was highest at 12 h under stress and then decreased. In the roots, the expression of PtSRO1b, PtSRO1c, and PtSRO2c was up-regulated with treatment and reached a peak at 24/36 h. In contrast, the expression of PtSRO2a and PtSRO2b showed a continuous decrease with treatment. To verify the accuracy of RNA-Seq data, we also conducted RT-qPCR validation of the nine genes under 150 mM salt stress at 24 h time point. As shown in Figure 7, the expression trend quantified by RT-PCR was consistent with the results profiled by RNA-Seq.

### 2.5. Analysis of Upstream TF Regulation Network

**Figure 8:**
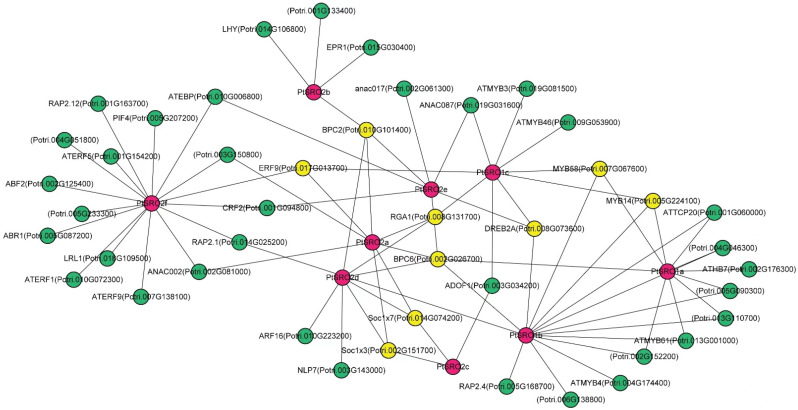
Transcriptional regulatory networks involving PtSROs. Pink nodes represent PtSROs, yellow nodes represent TFs with high connectivity with PtSROs, and green nodes represent proteins that may interact with PtSROs.

To better understand the underlying function of PtSROs, their upstream regulatory genes were predicted based on online websites and transcriptome data. As shown in Figure 8, a total of nine TFs with high connectivity were predicted to have direct interaction with PtSROs, and each TF regulates at least three PtSROs. Based on relevant studies, the TFs are closely related to stress responses in plants. For example, MYB14 and MYB58 are essential in regulating plant growth, development, metabolism, and adversity responses. MYB14 can be induced by exogenous IAA and drought stress, which has been proven to play an active role in mitigating drought-stressing damage [22]. Stress resistance of plants is closely linked to secondary cell wall formation and lignin deposition. MYB58 can directly activate lignin biosynthesis during secondary wall formation [22,30,31], which indicates its function in stress resistance indirectly. As ERF family members, DREB2A can be induced under conditions of dehydration and high salt stress in Arabidopsis [32], and the expression pattern of ERF9 was also closely correlated with salt stress [33]. Soc1x7 and Soc1x3 are members of the MADS-box family, which can improve drought tolerance by affecting the flowering period and stomatal opening [34,35]. RGA1 mutants in rice have strong drought tolerance [36]. BPC is a part of the complex network of TFs involved in cytokinin response. The knockout mutants of BPC2 enhance the osmotic tolerance of Arabidopsis seedlings [37]. Using ChIP-Seq, the direct target genes of BPC6 were identified in vivo, which contain the AtSRO family member, AtRCD1 [38]. 

### 2.6. RT-qPCR Validation of PtSROs under Different Stresses

**Figure 9:**
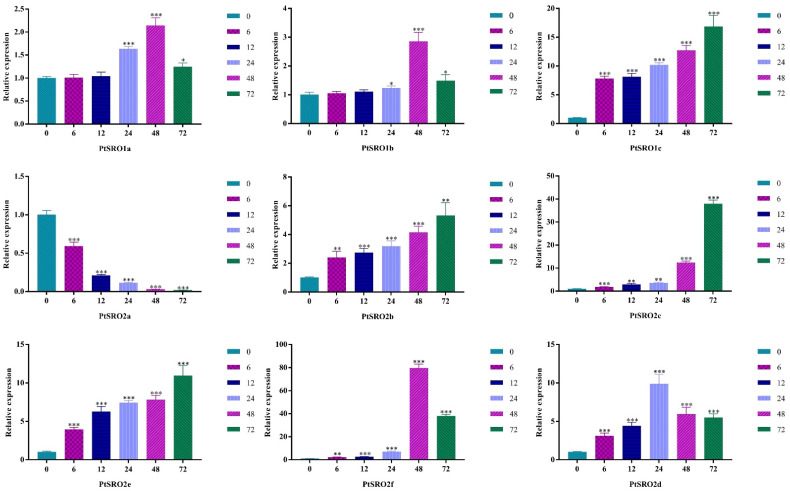
Expression patterns of PtSROs in response to NaCl. X-axis shows stress treatment time points; Y-axis represents the relative expression level. The data was processed using the 2^−ΔΔCt^ method. Transgenic lines and wild-type lines have significant differences indicated by asterisks in the error bars. (*t* test, * *p* < 0.05 ** *p *< 0.01 *** *p *< 0.001).

**Figure 10:**
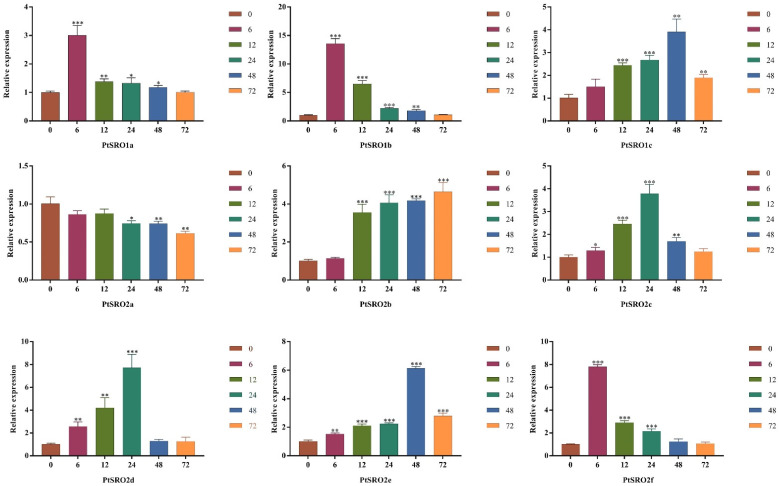
Expression patterns of PtSROs in response to PEG-6000. X-axis shows stress treatment time points; Y-axis represents relative expression levels. The data was processed using the 2^−ΔΔCt^ method. Transgenic lines and wild-type lines have significant differences indicated by asterisks in the error bars. (*t* test, * *p* < 0.05 ** *p *< 0.01 *** *p *< 0.001).

**Figure 11:**
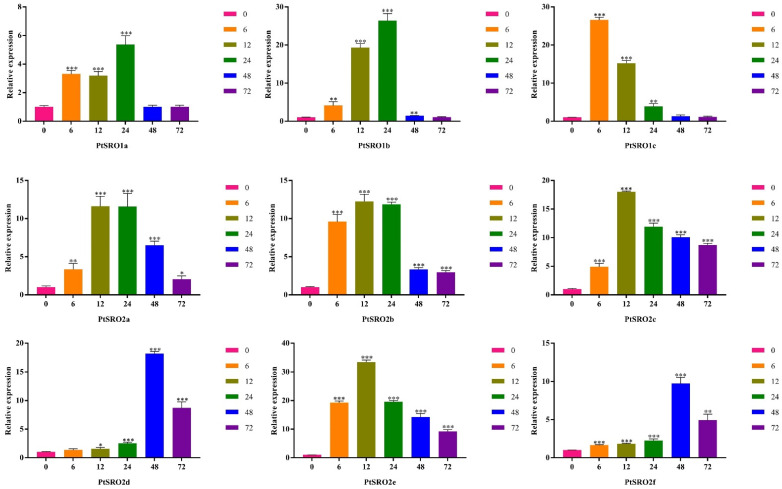
Expression patterns of PtSROs in response to ABA. X-axis shows stress treatment time points; Y-axis represents relative expression levels. The data was processed using the 2^−ΔΔCt^ method. Transgenic lines and wild-type lines have significant differences indicated by asterisks in the error bars. (*t* test, * *p* < 0.05 ** *p *< 0.01 *** *p *< 0.001).

To further explore the expression pattern of the nine PtSROs under stress conditions, RT-qPCR was used to quantify their relative expression levels in the leaves at different time points (0, 6, 12, 24, and 72 h) under 100 mΜ ABA, 150 mM NaCl, and 20% PEG, respectively. As Figure 9, Figure 10, Figure 11 and Table S6 show, each gene responded to stress conditions at different degrees, and expression trends of the genes in the same groups were similar. 

According to Figure 9, the expression pattern of group I members, including PtSRO1a and PtSRO1b, was similar under salt stress, which increased first and then decreased and peaked at 48 h. The expression of IIa members was up-regulated except PtSRO2a, which displayed a decreased trend with treatment. Members in groups IIb and Ic showed different expression trends with treatment under stress conditions. The relative expression levels of PtSRO2b, PtSRO2c, and PtSRO2e in the IIa group were up-regulated, while the expression of PtSRO2a was decreased. The relative expression level of PtSRO2d increased first and then decreased and reached a peak at 24 h. The relative expression level of PtSRO2f in group IIb also displayed an increase first and then a decreasing trend, where expression was highest at 48 h.

Under the PGE-6000 treatment (Figure 10), the expression level of group Ia members was highest at 6 h. In group IIa, PtSRO2a and PtSRO2b showed an opposite expression pattern, with a decreased and increased trend, respectively. While the expression of PtSRO2c and PtSRO2d reached a peak at the 24 h time point. In group IIb, the relative expression level of PtSRO2e and PtSRO2f was highest at 48 and 6 h, respectively. The expression pattern of PtSRO1c was similar to PtSRO2e.

As shown in Figure 11, all the PtSROs members showed similar expression patterns under ABA treatment, which increased first and then decreased. For example, group Ia showed a peak at 24 h. PtSRO2a, PtSRO2c, and PtSRO2b in group IIa all peaked at 12 h, while the expression of PtSRO2d was highest at 48 h. The maximum expression level of PtSRO2e and PtSRO2f in group IIb showed at 12 and 48 h, respectively. 

### 2.7. Subcellular Localization and Transcription Activating Activity of PtSROs

**Figure 12:**
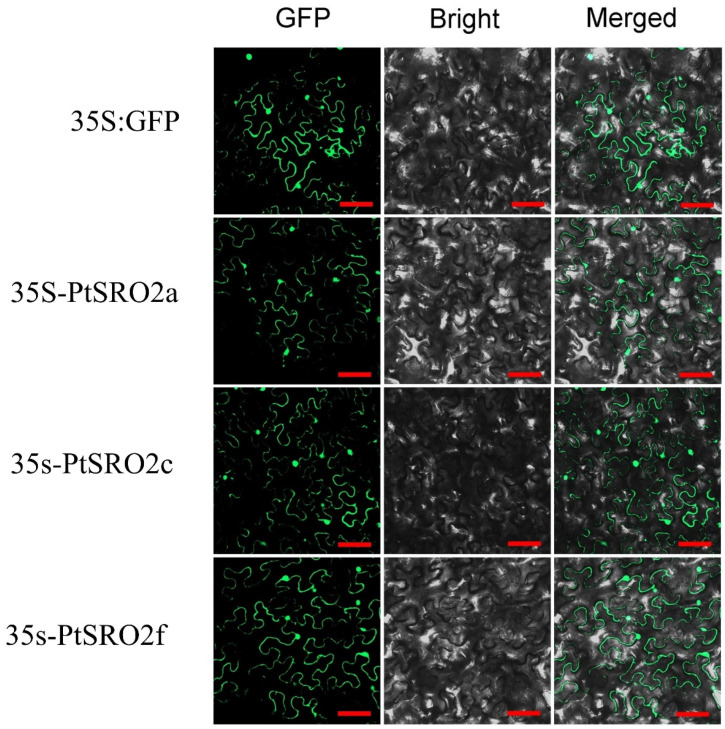
Subcellular localization of PtSRO2a, PtSRO2c, and PtSRO2f proteins. Using Agrobacterium-mediated transient expression, the control (35S-GFP) and fusion vectors (35S-PtSRO2a-GFP, 35S-PtSRO2c-GFP, 35S-PtSRO2f-GFP) were expressed in N. benthamiana leaves separately. Scale bar = 20 um.

Subcellular localization of PtSRO proteins was predicted using WoLF PSORT and Plant-mPL [39]. The prediction results indicated that the genes could be localized to the chloroplast, nucleus, and cytoplasm. We conducted transient expression assays of randomly selected three genes, including PtSRO2a, PrtSRO2c, and PtSRO2f, by injecting them into Nicotiana benthamiana. They were proven to be expressed in the nucleus and plasma membrane (Figure 12), consistent with their prediction results. 

We also conducted a yeast assay to validate the transcription-activating activity of PtSROs. We inserted coding sequences of *PtSRO2a*, *PtSRO2c*, and *PtSRO2f* into pGBKT7 in vivo and transformed them into Y2H with negative and positive controls, respectively. The positive control grew normally and turned blue on the SD/-Trp/-His/X-a-Gal medium, while pGBKT7-PtSRO2a, pGBKT7-PtSRO2c, pGBKT7-PtSRO2f and the negative control did not grow, indicating that PtSRO2a, PtSRO2c, and PtSRO2f protein has no self-activation (Figure S1).

## 3. Discussion

Plants survive in complex and diverse environments by regulating a large number of stress-responsive and structural genes, resulting in many physiological and metabolic processes [30]. As a small family unique to plants, SROs are key stress-responsive genes involved in various abiotic and oxidative stress responses in plants [21]. Although a growing number of SROs have been identified from various plants, their functions remain restricted to a few model plants, such as *RCD1*, *SRO1,* and *SRO5* in *Arabidopsis*. These studies confirm the functions of SROs in response to abiotic stresses and hormones. However, the underlying functions of SROs are undiscovered in poplar [16,31,40].

In our study, the results of the evolutionary tree analysis showed that PtSROs could be divided into group I and II. Group I can be divided into four subsets, Ia-Id, and Group II into two subsets, IIa and IIb (Figure 1). This corroborates with the classification results of SRO families reported in *Arabidopsis* earlier (*Arabidopsis* SIMILAR TO RCD-ONE genes are ubiquitous and respond to multiple abiotic stresses through diverse signaling pathways). In addition, the results of gene structure and protein motif analysis showed that SROs from the same group had similar gene structure and motif characteristics (Figure 2). This corresponds to the evolutionary tree results that we obtained. Collinearity analysis showed that two pairs of paralogous homologs formed as a result of gene duplication events found within the species (Figure 3). Cross-species collinearity analysis showed that PtSROs were homologous in *S. purpurea*, *E. grandis*, *Arabidopsis*, *G. hirsutum*, *O. sativa*, and *S. italicas*, with the highest number of homologous genes present in S. purpurea at eight. This may be because both are woody plants (Figure 4). Overall, the results of our bioinformatic analyses point to similarities between the poplar SRO gene family in terms of gene evolution and structural features to several species, such as Arabidopsis, suggesting that PtSROs may also be functionally similar to SROs in these species.

In the results of the PtSROs promoter analysis, all PtSROs promoters were found to contain a large number of stress-responsive and hormone-responsive elements (Figure 6 and Supplementary Table S5). Therefore, we subjected the plant material to different treatments, including salt, PEG, and ABA. The qPCR results for the three treatments showed that all PtSROs were involved in response to salt, PEG-6000, and ABA stresses. This suggests that PtSROs may act as key functional genes in the abiotic stress response pathway. In addition, subcellular localization indicated PtSRO2a, PtSRO2c, and PtSRO2f were all expressed in the nucleus, which provides the possibility that they can regulate other responsive genes. We also found that they have no self-activating activity, suggesting that they may require interactions with other regulatory proteins, such as TFs, to function. They may shuttle through the cytoplasm into the nucleus to interact with TFs when exposed to external stimuli, forming a regulatory network in response to abiotic stresses. Therefore, we further predicted the TFs that are upstream of PtSROs and genes that may interact with PtSROs (Figure 8). We found multiple transcription factors in the transcriptional regulatory network in which PtSROs have been reported in different species. For example, *RCD1-DREB2A* interactions play an important role in high salt and dehydration stress in Arabidopsis thaliana [10]. Our network has three PtSROs linked to DREB2A, of which PtSRO1b and AtRCD1 are from the same subset in the evolutionary tree and are more closely related (Figure 1). In addition, the *PtSRO1b* promoter contains multiple dehydration response elements. It is likely that PtSRO1b and DREB2A also interact in poplar species and are thus involved in salt and dehydration stresses. In the Arabidopsis BPC family study, ChIP-seq analysis of BPC6 transgenic plants revealed that AtRCD1 was one of the direct targets of BPC6 and that this pathway is involved in responding to cytokinins [39]. In our network, five linked genes exist in BPC6, of which PtSRO1a and PtSRO1b are most closely related to AtRCD1 evolution (Figure 1). Thus, in poplar, PtSRO1a and PtSRO1b may be similarly regulated by BPC6 and thus involved in the abiotic stress response. BPC2, also from the BPC family, shows enhanced salt tolerance in knockout strains in *Arabidopsis* [38]. In our network, there are four PtSROs linked to BPC2, PtSRO2a, PtSRO2b, PtSRO2d, and PtSRO2e, all of which are involved in salt stress response (Figure 9). It is suggested that these four PtSROs may be activated in response to salt stress through regulation by BPC2. PtSRO1a, PtSRO1b, and PtSRO1c act as linked genes in the network for MYB14 and MYB58, promoter regions that contain a large number of hormone-responsive and stress-responsive elements. MYB14 has been reported to be involved in growth hormone and drought response [22]. MYB58 has been reported to be involved in activating lignin biosynthesis, and the stress resistance of plants is closely linked to secondary cell wall formation and lignin deposition [22,32,40]. We hypothesize that PtSRO1a, PtSRO1b, and PtSRO1c are activated in response to abiotic stresses possibly regulated by MYB14 and MYB58. In our network, PtSRO1c, PtSRO2a, and PtSRO2d are linked to RGA1. PtSRO2a, PtSRO2c, and PtSRO2d are linked to Soc1x7 and Soc1x3. PtSRO1c, PtSRO2a, PtSRO2c, and PtSRO2d promoter regions contain multiple dehydration and drought response elements, and all four PtSROs are involved in PEG stress response (Figure 10). The RGA1, Soc1x7, and Soc1x3 linked to them have been reported to be involved in drought stress [35,36,37], suggesting that these four PtSROs may be induced to participate in PEG stress by RGA1, Soc1x7, and Soc1x3. The NAC family is well known as a high-level regulator in the plant transcriptional regulatory network. In rice studies, NAC1 genes were found to have protein interactions with OsSRO1, and they, in turn, are involved in response to drought and oxidative stresses [16]. In our network, multiple NAC-SRO chains were identified, including ANAC002-PtSRO2f, ANAC002-PtSRO2a, ANAC017-PtSRO2e, ANAC087-PtSRO2e and ANAC087-PtSRO1c (Figure 8). These SROs may be induced to respond to drought stress after interacting with NAC family proteins. In conclusion, both the results of our promoter analysis and the gene expression following adversity stress clarify that the PtSRO family may act as one of the key functional gene families in the abiotic stress response pathway of plants.

## 4. Materials and Methods

### 4.1. Identification of SROs in Poplar

Amino acid sequences of all poplar SROs were extracted from the Phytozome database (https://phytozome-next.jgi.doe.gov/ *Populus trichocarpa* v4.1, accessed on 15 October 2023), and conserved structural domains of WWE structural domain (PF02825) were identified by Hidden Markov Model (HMM) profiling. PARP structured domain (PF00644) and the RST structured domain (PF12174) were obtained from the Pfam database (http://pfam.xfam.org/, accessed on 16 October 2023) [41]. HMMER 3.0 [42] and SMART databases (http://SMART.embleidelberg.de/, accessed on 16 October 2023) were used to screen potential SRO proteins in *Populus*, *Salix purpurea*, *Setaria italica*, and *Triticum aestivum*. The physical and chemical parameters of SRO proteins were calculated using the ExPASy website (http://web.expasy.org/protparam/, accessed on 16 October 2023). 

### 4.2. Phylogenetic Analysis of PtSROs

SRO proteins in *Arabidopsis*, *Salix purpurea*, *Setaria italica*, and *Triticum aestivum* were obtained from Phytozome and TAIR website (https://www.arabidopsis.org/ accessed on 16 October 2023). All identified SRO proteins were used for multiple sequence alignment with ClustalX 1.83 [43]. The neighbor-joining (NJ) method was used to construct a phylogenetic tree (1000 bootstrap replications) in MEGA 5.0 software [26].

### 4.3. Chromosome Distribution and Covariance Analysis of PtSROs 

Poplar genomic data downloaded from the Phytozome database were used to map each SRO gene to its corresponding chromosomal location based on their positional information with TBtools [28]. MCScanX (Multicollinearity Scanning Toolkit) software was used to determine their covariance relationships, which were visualized using TBtools [27,28].

### 4.4. Analysis of Gene Structure and Conserved Motifs of PtSROs

Poplar genome sequences were also downloaded from the Phytozome database. GSDS (http://GSDS.cbi.pku.edu.cn/, accessed on 20 October 2023) was used to analyze the gene structure of SRO family members. Conservative modes were analyzed using the online software MEME (http://meme-suite.org/tools/meme, accessed on 20 October 2023) with default parameters and visualized using TBtools [28,44].

### 4.5. Analysis of Cis-Acting Elements in the Promoter Regions of PtSROs

The 2000 bp upstream sequences of PtSROs were extracted from the Phytozome database. Cis-elements were predicted with PLACE (https://www.dna.affrc.go.jp/PLACE/place_seq.shtml, accessed on 23 January 2023) and visualized with TBtools [28,45].

### 4.6. Upstream TF Regulation Network Analysis

Predictions of upstream regulators of *PtSROs* were performed using the PlantRegMap website (http://plantregmap.gao-lab.org/go.php, accessed on 22 October 2023) [46]. Expression information of upstream genes was obtained from transcriptome data (SRP267437). Pearson correlation coefficients between upstream genes and *SROs* were calculated, and significantly related gene pairs were screened using a threshold of |R| ≥ 0.8 and *p* ≥ 0.1. Networks were plotted using Cytoscape (https://cytoscape.org/download.html, accessed on 22 October 2023). 

### 4.7. Plant Material, Growth Conditions, and Stress Treatments

In this study, haploid *Populus simonii × Populus nigra* seedlings were grown at 26 °C with a 16 h light /8 h dark cycle in Northeast Forestry University’s experimental field. One-month seedlings were treated with 20% PEG-6000, 150 mM NaCl, and 100 mM abscisic acid (ABA) solution for 0, 6, 12, 24, 48, and 72 h, respectively [3,47,48]. Three biological repeats were prepared for each sample at different time points with treatments. RNA was extracted from all harvested samples by freezing them rapidly in liquid nitrogen. 

### 4.8. RNA-Seq

The leaves and root tissues of *Populus simonii × Populus nigra* under 150 mM salt stress at 0, 12, 24, and 36 h with respective 4 biological repeats were sent to Novogene company (https://en.novogene.com/, accessed on 10 October 2023) for RNA-Seq. Data processing and gene expression profiling were conducted according to our previous study [48].

### 4.9. Expression Pattern Analysis Using qRT-PCR

To verify the expression pattern of *SROs* in *Populus simonii × Populus nigra* under abiotic stresses, we quantified their relative expression levels under stress conditions using qRT-PCR as described in our previous study [49]. The relative expression was calculated using the 2^−ΔΔCt^ method, with the Actin gene as the internal reference gene. All primers were listed in Supplementary Table S7.

### 4.10. Subcellular Localization of PtSRO2a, PtSRO2c, PtSRO2f

Subcellular localization of PtSRO proteins was predicted using WoLF PSORT (https://wolfpsort.hgc.jp/, accessed on 12 October 2023) and Plant-mPLoc (http://www.csbio.sjtu.edu.cn/bioinf/plant-multi/, accessed on 12 October 2023). The full-length coding sequences of *PtSRO2a*, *PtSRO2c,* and *PtSRO2f* without stop codon were fused into 35S promoter-driven pBI-121-GFP vectors. The fusion vectors (35S-*SRO2a*-GFP, 35S-*SRO2c*-GFP, 35S-*SRO2f*-GFP) and 35S-GFP as positive controls were transferred into *Agrobacterium tumefaciens* GV3101 using liquid nitrogen freeze-thaw method. The Agrobacterium tumefaciens containing fusion vectors were injected into the leaves of one-month-old *N. benthamiana* seedlings by transient transformation. Then the tobacco leaves were incubated in the dark for 48 h and observed under LMS800 laser confocal microscope (The machine comes from Shanghai Saita Industrial Co., Ltd., Shanghai, China).

### 4.11. Detection of Transcriptional Activation Activity of PtSRO2a, PtSRO2c, and PtSRO2f

In order to generate a fusion vector containing the GAL4-DNA structural domain, the full-length coding sequences of PtSRO2a, PtSRO2c, and PtSRO2f were inserted into pGBKT7, respectively. The three recombinant fusion vectors, the pGBKT_7 vector (negative control) and pGBKT-53/pGADT7-T (positive control), were transferred into yeast two-hybrid (Y2H) cells, respectively. The cells were grown on SD/-Trop, SD/-Trop/-His, and X-a-Gal media for 3–5 days at 30 °C.

## 5. Conclusions

In this study, a total of nine *SRO* members were identified from *Populus simonii × Populus nigra*, which can be divided into two groups. The nine *PtSROs* were randomly distributed on six chromosomes in poplar. Cis-element analysis showed there were many cis-regulatory elements related to abiotic stress and hormone-induced elements in the promoter regions of *PtSROs*. Expression pattern analysis indicated that *PtSROs* could be induced in the leaves and roots by osmotic, salt, and ABA treatments. The relative expression levels of *PtSROs* were higher in the leaves than those in the roots. In addition, the nine genes displayed different expression trends in the two tissues, reaching a peak at different time points under different stresses. In particular, *PtSRO1c* and *PtSRO2c* were more prominent in response to stress treatments. Moreover, we screened a total of nine upstream regulatory TFs of *PtSROs*, and each TF was predicted to regulate at least three *PtSROs*. Functional annotation indicated that all nine TFs were involved in abiotic stress responses. Similarly, *PtSRO1c* and *PtSRO2c* had more predicted reciprocal TFs. The study provides a theoretical basis for functional identification of the *SRO* family gene in abiotic stress in poplar.

## Figures and Tables

**Table 1:** The Ka/Ks ratios of duplication for duplicate SRO genes in poplar.

Duplicated Gene Pairs	Ka	Ks	Ka/Ks	The Length of Homologous Fragment/Bp	Homology	Duplicationdatc (MYA)
PtSRO1a/PtSRO1b	0.714519	0.2281039	0.3132428	731	88%	7.603463333
PtSRO2e/PtSRO2f	0.0873593	0.2545988	0.3431254	1223	90%	8.486626667

## Data Availability

Not applicable.

## Supplementary Materials

The supporting information can be downloaded at: https://www.mdpi.com/article/10.3390/ijms24044146/s1.
