# Supplementary figures and images for: Genome-Wide Analysis of SIMILAR TO RCD ONE (SRO) Family Revealed Their Roles in Abiotic Stress in Poplar
Source: Int J Mol Sci. 2023 Feb 19;24(4):4146. doi: 10.3390/ijms24044146 (PMC9961671; doi:10.3390/ijms24044146)

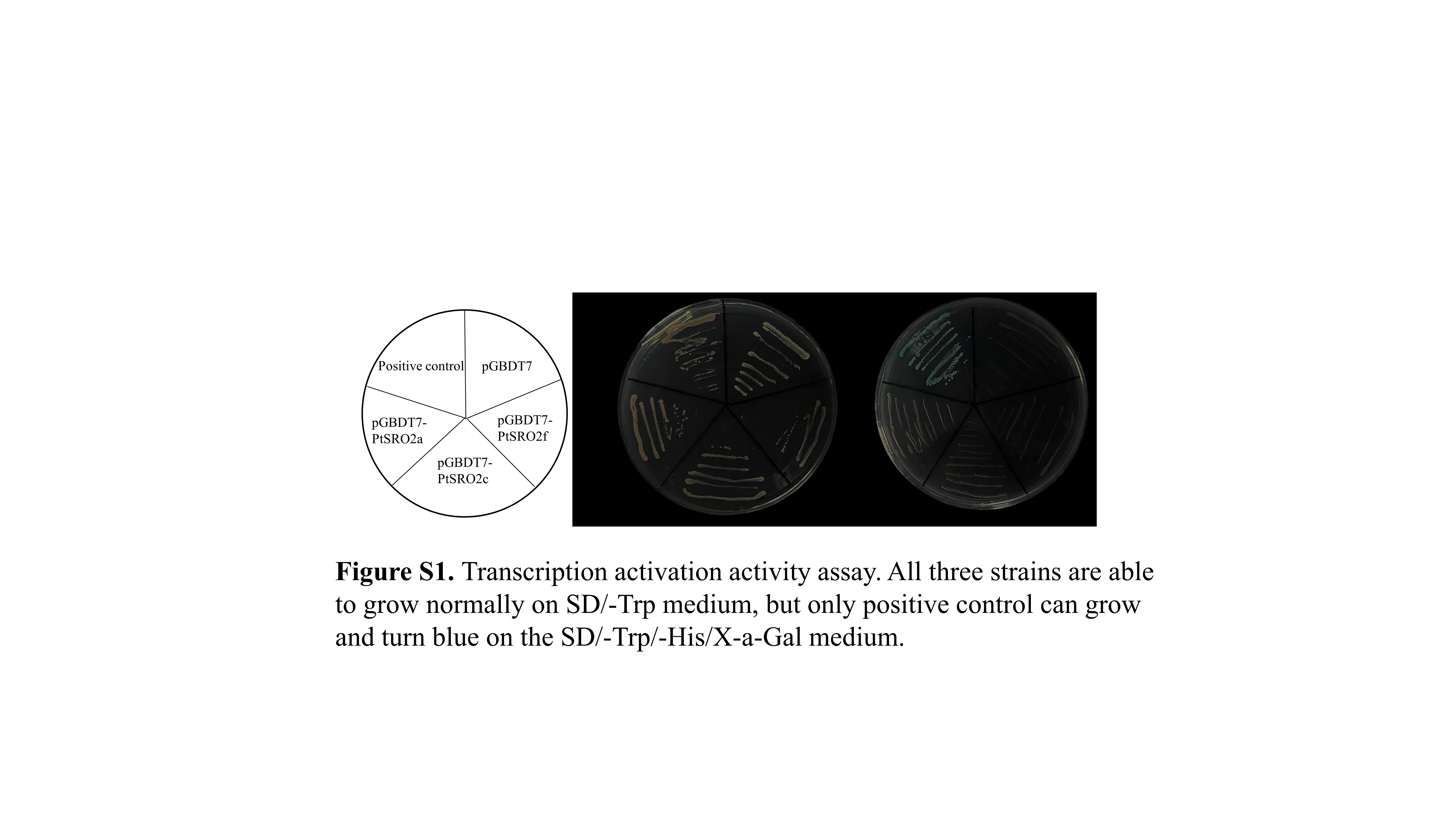

Supplement: Supplementary file 1 [file ijms-24-04146-s001.zip › Figure S1. Transcription activation activity assay.jpg]
